# Insulin lispro low mixture twice daily vs basal insulin glargine once daily and prandial insulin lispro once daily as insulin intensification strategies in patients with type 2 diabetes: A Latin American subpopulation analysis of a randomized trial

**DOI:** 10.1186/1758-5996-7-S1-A57

**Published:** 2015-11-11

**Authors:** Douglas Eugenio Barbieri, Ran Duan, Jorge Gross, Bruno Linetzky, Janaina Martins De Lana, Arturo Rojas, Georgina Sposetti, Oded Stempa, Angel Rodriguez

**Affiliations:** 1 Universidade Federal de São Paulo, São Paulo, Brazil

## Background/aim

This post-hoc analysis examined the efficacy and safety of twice-daily insulin lispro low mixture (LM25) and once-daily basal insulin glargine plus once-daily prandial insulin lispro (IGL) in a Latin American subpopulation (Argentina, Brazil, and Mexico) of participants with type 2 diabetes mellitus (T2D).

## Materials and methods

This phase 4, randomized, open-label, parallel-arm trial included participants aged 18–75 yrs. with T2D who were taking once-daily insulin glargine and stable doses of metformin and/or pioglitazone and had glycosylated hemoglobin (HbA1c) between ≥7.5% and ≤10.5% and fasting plasma glucose ≤6.7 mmol/L (121 mg/dL). Participants were randomized 1: 1 to receive twice-daily LM25 (before breakfast and dinner) or basal insulin glargine (at bedtime) and IGL (before the largest daily meal) in addition to their existing dose of metformin and/or pioglitazone for 24 weeks. The primary efficacy outcome was the change in HbA1c from baseline to Week 24.

## Results

**Figure 1:**
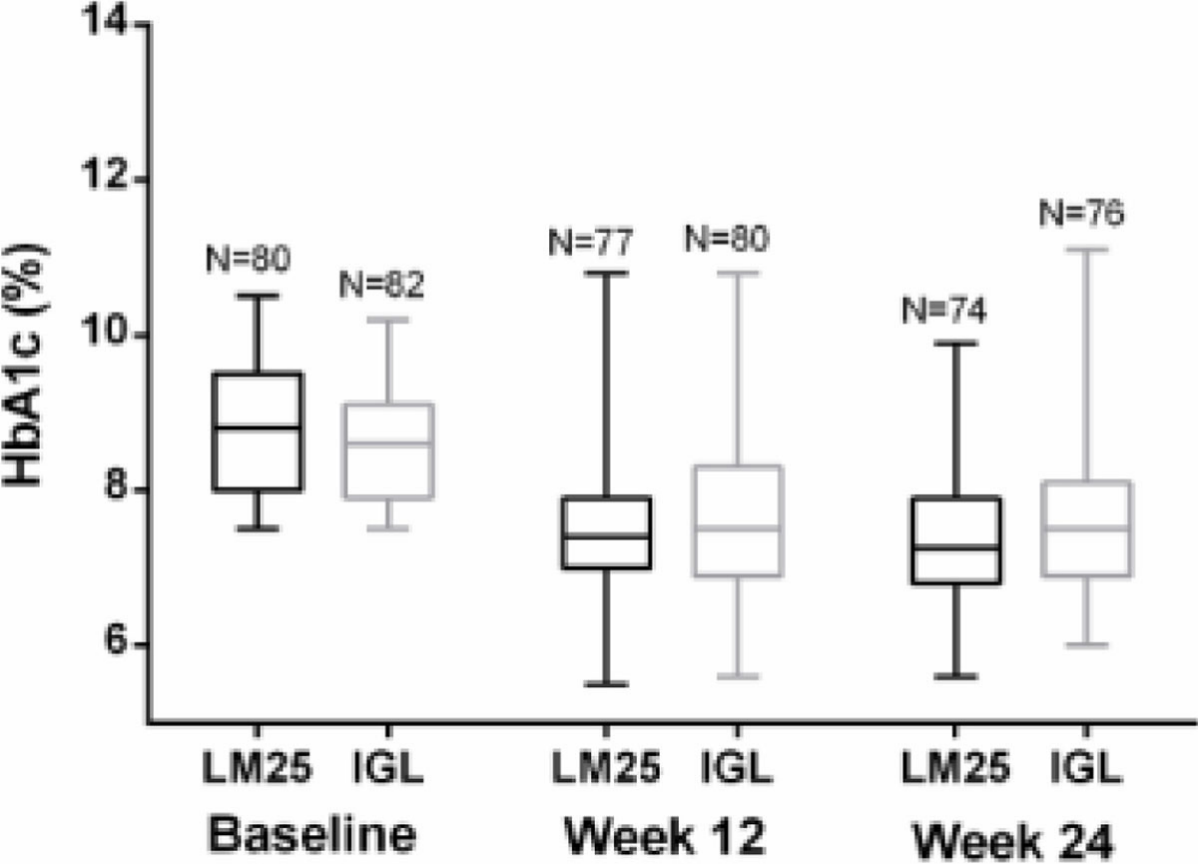
Observed HbA1c levels at Baseline, Week 12, and Week 24 in patients receiving insulin lispro low mixture (LM25; 75% insulin lispro protamine suspension and 25% insulin lispro solution 25%) twice daily or basal insulin glargine once daily and prandial insulin lispro once daily (IGL). Abbreviation: HbA1c=glycosylated hemoglobin.

A total of 162 participants (80 LM25; 82 IGL) with a mean (standard deviation [SD]) age of 57.3 (9.0) yrs. and body mass index of 31.3 (5.2) kg/m2 were included. The mean (SD) change in HbA1c (%) from baseline was -1.5 (1.0) in the LM25 group and -1.0 (1.2) in the IGL group (Figure 1). At Week 24, 35.1% of participants in the LM25 group and 31.6% of participants in the IGL group achieved the target HbA1c <7.0%. Fasting blood glucose and glycemic variability at Week 24 were similar between the 2 groups, as was the mean (SD) total daily insulin dose (LM25=61.0 [27.6] IU; IGL=60.6 [24.3] IU). The mean (SD) rate of total hypoglycemia per 30 days was numerically similar between the two groups (LM25=1.6 [2.2]; IGL=1.8 [2.6] [overall study period]). Mean (SD) weight gain from baseline to Week 24 was 2.4 (2.9) kg in the LM25 group and 1.0 (3.1) kg in the IGL group. Treatment-emergent adverse events were similar between the 2 groups.

## Conclusions

The results of this post-hoc analysis in a Latin American population are consistent with the results reported in the trial-level population and suggest that both LM25 and IGL are viable treatment options for insulin intensification in patients with T2D who do not achieve glycemic control on basal insulin glargine. ClinicalTrials.gov Number: NCT01175824.

